# Supplementary material for: Inferring rates of metastatic dissemination using stochastic network models
Source: PLoS Comput Biol. 2019 Apr 1;15(4):e1006868. doi: 10.1371/journal.pcbi.1006868 (PMC6459558; doi:10.1371/journal.pcbi.1006868)
Supplement: S1 Data — (DOCX) [file pcbi.1006868.s001.docx]

| ***TNM Classification of tumour*** |  |
| --- | --- |
| ***T-stage, n (%)*** |  |
| 1 | 17 (12) |
| 2 | 59 (42) |
| 3 | 23 (16) |
| 4 | 43 (30) |
| ***N-stage, n (%)*** |  |
| 0 | 63 (44) |
| 1 | 41 (29) |
| 2 | 34 (24) |
| 3 | 4 (3) |
| ***M-stage, n (%)*** |  |
| 1 | 2 (1)* |
| 0 | 140 (99) |
|  |  |
| ***Oncological treatment, n (%)*** |  |
| Surgery only | 3 (2) |
| Surgery + radiotherapy | 68 (48) |
| Surgery + radiotherapy + chemotherapy | 7 (5) |
| Radiotherapy, radical intent | 12 (8) |
| Radiotherapy + chemotherapy, radical intent | 32 (23) |
| Radiotherapy, palliative intent | 18 (13) |
| No treatment | 2 (1) |

## **Supplementary material: S1 Clinical data of the tongue cancer cohort**

** Both* *patients had* *metastasis to the lung.*

### Treatment

For stage I and II tumors surgery (partial resection or hemiglossectomy) was the main treatment of choice, provided that the tumor was judged to be resectable with proper margins. In most cases supraomohyoid neck dissection was also performed. In cases with positive or close surgical margins or positive nodes, adjuvant treatment was applied, either as radiotherapy or as chemoradiotherapy.

Stage III and IV tumors were treated either with primary surgery as described above followed by adjuvant radiotherapy or with chemoradiotherapy with curative intent. Radiotherapy with curative intent was given with conventional fractionation, 2 Gy (Gray)/fraction to a final dose of 68 Gy, alternatively with hyperfractionation, 1.7 Gy/fraction twice daily to a final dose of 64.6 Gy. Twenty-nine patients received brachytherapy in addition to external radiotherapy. Chemotherapy was given either as induction therapy (cisplatin + fluorouracil (5-Fu)) or concomitant to radiotherapy (cisplatin once weekly).

In cases with advanced tumors or patients with poor performance status, high age and/or severe comorbidity, radiotherapy with palliative intent was performed. Two patients received neither surgery nor radiotherapy nor chemotherapy. One of these patients declined treatment; the other received best supportive care.
