# Supplementary material for: Inferring rates of metastatic dissemination using stochastic network models
Source: PLoS Comput Biol. 2019 Apr 1;15(4):e1006868. doi: 10.1371/journal.pcbi.1006868 (PMC6459558; doi:10.1371/journal.pcbi.1006868)
Supplement: S2 Data — (DOCX) [file pcbi.1006868.s002.docx]

## **Supplementary material: S2 Data for tumour stage and metastatic spread**

Each row in the below table corresponds to a patient in the cohort. The first column contains the primary tumour stage at diagnosis and the following columns show if the patient was positive (=1) or negative (=0) for metastases in lymph node stations I-IV.

| Tumour stage | Station I | Station II | Station III | Station IV |
| --- | --- | --- | --- | --- |
| 4 | 1 | 0 | 0 | 0 |
| 3 | 0 | 0 | 0 | 0 |
| 4 | 0 | 0 | 0 | 0 |
| 1 | 0 | 1 | 0 | 0 |
| 2 | 0 | 0 | 0 | 0 |
| 2 | 0 | 0 | 0 | 0 |
| 1 | 0 | 1 | 0 | 0 |
| 3 | 0 | 1 | 1 | 0 |
| 4 | 1 | 1 | 1 | 0 |
| 4 | 0 | 0 | 0 | 0 |
| 3 | 0 | 0 | 0 | 0 |
| 4 | 0 | 0 | 0 | 0 |
| 1 | 0 | 0 | 0 | 0 |
| 3 | 0 | 0 | 0 | 0 |
| 2 | 0 | 0 | 0 | 0 |
| 2 | 0 | 1 | 0 | 0 |
| 4 | 0 | 0 | 1 | 0 |
| 2 | 0 | 0 | 0 | 0 |
| 4 | 0 | 0 | 0 | 0 |
| 3 | 1 | 1 | 1 | 0 |
| 2 | 0 | 0 | 0 | 0 |
| 2 | 0 | 0 | 0 | 0 |
| 4 | 1 | 1 | 1 | 0 |
| 1 | 0 | 0 | 1 | 0 |
| 2 | 0 | 0 | 0 | 0 |
| 4 | 0 | 0 | 0 | 0 |
| 3 | 0 | 0 | 0 | 0 |
| 4 | 0 | 1 | 0 | 0 |
| 2 | 0 | 0 | 0 | 0 |
| 3 | 1 | 0 | 0 | 0 |
| 1 | 0 | 1 | 1 | 0 |
| 2 | 0 | 0 | 0 | 0 |
| 1 | 0 | 0 | 0 | 0 |
| 2 | 0 | 0 | 0 | 0 |
| 2 | 0 | 1 | 0 | 0 |
| 2 | 1 | 0 | 0 | 0 |
| 2 | 1 | 1 | 0 | 0 |
| 1 | 0 | 0 | 0 | 0 |
| 2 | 0 | 0 | 0 | 0 |
| 4 | 0 | 0 | 0 | 0 |
| 2 | 0 | 1 | 1 | 0 |
| 1 | 0 | 0 | 0 | 0 |
| 2 | 0 | 1 | 0 | 0 |
| 2 | 0 | 0 | 0 | 0 |
| 3 | 0 | 1 | 0 | 0 |
| 2 | 0 | 0 | 0 | 0 |
| 2 | 0 | 1 | 0 | 0 |
| 4 | 0 | 1 | 0 | 0 |
| 4 | 1 | 1 | 0 | 0 |
| 1 | 0 | 1 | 0 | 1 |
| 4 | 0 | 1 | 0 | 0 |
| 2 | 0 | 1 | 1 | 0 |
| 2 | 0 | 1 | 0 | 0 |
| 3 | 0 | 0 | 0 | 0 |
| 2 | 1 | 1 | 0 | 0 |
| 2 | 0 | 0 | 0 | 0 |
| 3 | 0 | 0 | 0 | 0 |
| 1 | 1 | 0 | 0 | 0 |
| 4 | 1 | 1 | 1 | 1 |
| 4 | 0 | 1 | 1 | 0 |
| 2 | 0 | 0 | 0 | 0 |
| 4 | 1 | 1 | 1 | 0 |
| 2 | 1 | 1 | 0 | 0 |
| 4 | 0 | 1 | 0 | 0 |
| 1 | 0 | 0 | 0 | 0 |
| 3 | 0 | 0 | 1 | 1 |
| 2 | 0 | 0 | 0 | 0 |
| 2 | 0 | 1 | 0 | 0 |
| 4 | 0 | 0 | 0 | 0 |
| 4 | 1 | 1 | 0 | 0 |
| 2 | 0 | 1 | 0 | 0 |
| 2 | 1 | 0 | 0 | 0 |
| 1 | 0 | 0 | 0 | 0 |
| 3 | 1 | 0 | 0 | 0 |
| 4 | 1 | 1 | 0 | 0 |
| 2 | 1 | 0 | 0 | 0 |
| 2 | 0 | 0 | 0 | 0 |
| 4 | 0 | 0 | 0 | 0 |
| 1 | 0 | 0 | 1 | 0 |
| 2 | 1 | 0 | 0 | 0 |
| 4 | 0 | 0 | 0 | 0 |
| 3 | 0 | 0 | 0 | 0 |
| 2 | 0 | 0 | 0 | 0 |
| 4 | 0 | 0 | 0 | 0 |
| 2 | 1 | 1 | 0 | 1 |
| 4 | 0 | 0 | 1 | 0 |
| 4 | 1 | 0 | 0 | 0 |
| 2 | 0 | 1 | 0 | 0 |
| 3 | 1 | 1 | 0 | 0 |
| 2 | 0 | 1 | 1 | 0 |
| 4 | 0 | 1 | 1 | 0 |
| 1 | 0 | 0 | 0 | 0 |
| 3 | 0 | 1 | 1 | 0 |
| 2 | 0 | 0 | 0 | 0 |
| 4 | 0 | 0 | 0 | 0 |
| 3 | 0 | 0 | 0 | 0 |
| 1 | 0 | 0 | 0 | 0 |
| 4 | 0 | 1 | 0 | 0 |
| 2 | 0 | 1 | 0 | 1 |
| 2 | 0 | 0 | 0 | 0 |
| 2 | 0 | 1 | 1 | 0 |
| 2 | 0 | 0 | 0 | 0 |
| 2 | 0 | 0 | 0 | 0 |
| 2 | 0 | 0 | 0 | 0 |
| 2 | 0 | 0 | 0 | 0 |
| 2 | 0 | 0 | 0 | 0 |
| 4 | 1 | 1 | 0 | 0 |
| 2 | 1 | 0 | 0 | 0 |
| 4 | 0 | 0 | 0 | 0 |
| 1 | 0 | 1 | 0 | 0 |
| 4 | 0 | 0 | 0 | 0 |
| 3 | 0 | 0 | 0 | 0 |
| 4 | 0 | 1 | 0 | 0 |
| 2 | 0 | 0 | 0 | 0 |
| 4 | 1 | 1 | 1 | 0 |
| 2 | 0 | 0 | 0 | 0 |
| 4 | 0 | 1 | 0 | 0 |
| 3 | 0 | 1 | 0 | 0 |
| 4 | 1 | 0 | 0 | 0 |
| 4 | 1 | 0 | 0 | 0 |
| 4 | 0 | 1 | 1 | 0 |
| 2 | 0 | 0 | 0 | 0 |
| 4 | 0 | 1 | 0 | 0 |
| 4 | 0 | 0 | 0 | 0 |
| 2 | 0 | 0 | 0 | 0 |
| 4 | 0 | 1 | 0 | 0 |
| 4 | 0 | 1 | 0 | 0 |
| 2 | 0 | 1 | 0 | 0 |
| 2 | 0 | 1 | 0 | 0 |
| 3 | 0 | 1 | 1 | 0 |
| 3 | 0 | 1 | 1 | 0 |
| 2 | 0 | 1 | 0 | 0 |
| 4 | 1 | 0 | 0 | 0 |
| 3 | 0 | 0 | 0 | 0 |
| 3 | 0 | 0 | 1 | 0 |
| 2 | 1 | 0 | 1 | 0 |
| 2 | 0 | 0 | 0 | 0 |
| 2 | 0 | 1 | 0 | 0 |
| 2 | 0 | 0 | 0 | 0 |
| 1 | 0 | 1 | 0 | 0 |
| 3 | 0 | 1 | 0 | 0 |
